# Retraction Note: The comparison of two different protocols ultra-long versus medroxyprogesterone acetate in women with ovarian endometriosis: a prospective randomized controlled trial

**DOI:** 10.1186/s12978-025-01977-4

**Published:** 2025-03-09

**Authors:** Haiyan Guo, Tong Du, Hongyuan Gao, Qianwen Xi, Ling Wu, Qifeng Lyu, Qianqian Zhu

**Affiliations:** Department of Assisted Reproduction, Shanghai Ninth People’s Hospital, Shanghai JiaoTong University School of Medicine, Center for Specialty Strategy Research of Shanghai Jiao Tong University China Hospital Development Institute, Shanghai, 200011 China

**Retraction Note: Reproductive Health (2022) 19:198** 10.1186/s12978-022-01500-z

The Editors-in-Chief have retracted this article because it overlaps significantly with another article [1] from the same author group. Haiyan Guo, Hongyuan Gao, Qianwen Xi, Ling Wu, Qifeng Lyu and Qianqiun Zhu have not responded to correspondence from the Publisher about this retraction. Tong Du has stated that they were not aware of the submission of this article and did not participate in this study.
